# Electrical Stimulation Promotes Regeneration of Defective Peripheral Nerves after Delayed Repair Intervals Lasting under One Month

**DOI:** 10.1371/journal.pone.0105045

**Published:** 2014-09-02

**Authors:** Chungui Xu, Yuhui Kou, Peixun Zhang, Na Han, Xiaofeng Yin, Jiuxu Deng, Bo Chen, Baoguo Jiang

**Affiliations:** Department of Trauma and Orthopaedics, Peking University People's Hospital, Beijing, P.R. China; Friedrich-Alexander University Erlangen, Germany

## Abstract

**Background:**

Electrical stimulation (ES) has been proven to be an effective means of enhancing the speed and accuracy of nerve regeneration. However, these results were recorded when the procedure was performed almost immediately after nerve injury. In clinical settings, most patients cannot be treated immediately. Some patients with serious trauma or contaminated wounds need to wait for nerve repair surgery. Delays in nerve repair have been shown to be associated with poorer results than immediate surgery. It is not clear whether electrical stimulation still has any effect on nerve regeneration after enough time has elapsed.

**Methods:**

A delayed nerve repair model in which the rats received delayed nerve repair after 1 day, 1 week, 1 month, and 2 months was designed. At each point in time, the nerve stumps of half the rats were bridged with an absorbable conduit and the rats were given 1 h of weak electrical stimulation. The other half was not treated. In order to analyze the morphological and molecular differences among these groups, 6 ES rats and 6 sham ES rats per point in time were killed 5 days after surgery. The other rats in each group were allowed to recover for 6 weeks before the final functional test and tissue observation.

**Results:**

The amounts of myelinated fibers in the distal nerve stumps decreased as the delay in repair increased for both ES rats and sham ES rats. In the 1-day-delay and 1-week-delay groups, there were more fibers in ES rats than in sham ES rats. And the compound muscle action potential (CMAP) and motor nerve conduction velocity (MNCV) results were better for ES rats in these two groups. In order to analyze the mechanisms underlying these differences, Masson staining was performed on the distal nerves and quantitative PCR on the spinal cords. Results showed that, after delays in repair of 1 month and 2 months, there was more collagen tissue hyperplasia in the distal nerve in all rats. The brain-derived neurotrophic factor (BDNF) and trkB expression levels in the spinal cords of ES rats were higher than in sham ES rats. However, these differences decreased as the delay in repair increased.

**Conclusions:**

Electrical stimulation does not continue to promote nerve regeneration after long delays in nerve repair. The effective interval for nerve regeneration after delayed repair was found to be less than 1 month. The mechanism seemed to be related to the expression of nerve growth factors and regeneration environment in the distal nerves.

## Introduction

Peripheral nerve injury is common in clinical settings [1]–[3]. Despite meticulous surgical techniques and many repair methods, full functional outcome, especially with respect to motor function, is rarely achievable [4]–[7]. Poor functional outcomes after repair of peripheral nerve lesions have been attributed to two main factors [8]. First, the proximal stump of the injured nerve grows slowly across the coaptation sites or nerve gaps. The nerve regeneration rate has been reported to be 1–3 mm/d [9]. However, there was a latent period of 1 month for all the axons to regenerate across the lesion site and form functional connections [10]. Second, there is a relatively short time frame for the support of regenerating axons after injury by the injured neurons and the denervated Schwann cells. Findings that nerve grafts lacking Schwann cells fail to support axonal regeneration demonstrated that Schwann cells were prerequisite for axonal regeneration [11], [12]. Long–term chronic denervation of Schwann cells dramatically reduced regeneration of axons into the distal nerve stump [13]. Any approach that overcomes these restrictions could improve the results of nerve regeneration after injury.

Electrical stimulation (ES) has been proved to be an effective method of enhancing the speed and accuracy of sensory and motor axonal regeneration. Previous studies have shown that ES applied immediately after surgical repair of the transected femoral nerve can promote the regeneration of all motor axons over a 25 mm distance from the surgical site as observed after 3 weeks. Without ES, the regenerating axons require 8–10 weeks to reach this level [14]. A later experiment was performed on the impact of ES on the regeneration of the sensory nerve. It was found that electrical stimulation lasting for 1 h led to significantly more DRG neurons regenerating into cutaneous and muscle branches than among animals treated with sham ES or with ES lasting for longer time [15], [16]. Accelerated growth and specificity of sensory and motor nerve regeneration were demonstrated to be related to increased expression of some growth factors, such as brain-derived neurotrophic factor (BDNF), its receptor trkB, growth-associated protein 43 (GAP-43), and similar factors [17], [18]. Electrical stimulation up-regulating the expression of these nerve growth factors leads to axonal growth in a faster mode. However in these experiments, the nerves were repaired and electrically stimulated immediately after nerve transection. In clinical settings, many patients with nerve injuries cannot be treated immediately after nerve injury [19], [20]. Some trauma patients suffer from severe nerve injuries or infection of the wound. In these cases, neural prosthesis must be delayed until the patient's physical condition improves. Whether electrical stimulation remains an effective means of improving nerve regeneration after delays in nerve repair and after how long a period are not known.

The objective of this study is to determine whether the application of 1 h of electrical stimulation can promote axonal regeneration after delayed nerve repair. Previous studies have shown that electrical stimulation of regenerating nerves initiated at day 8 after nerve repair had significantly more axons than controls receiving no ES [22]. However, in this study, percutaneous ES was used on the animals, which may not have stimulated the transected nerves effectively. In the current experiment, the sciatic nerve was electrical-stimulated directly after delays lasting different periods. The results of axonal regeneration of ES and sham ES rats in different groups were compared after 6 weeks' recovery. In order to analyze the mechanism involved, the distal nerves and spinal cords were also harvested for Masson staining and quantitative PCR shortly after the secondary surgery.

## Materials and Methods

### Ethics statement

All animal procedures were conducted under a protocol that was reviewed and approved by the Research Ethics Committee at PKUPH and met international biomedical ethics guidelines. Female Sprague-Dawley (SD) rats weighing 200–250 g were maintained under specific-pathogen-free laboratory conditions on a 12 h light/dark cycle with free access to pellet food and water. The SD rats were randomly separated into four groups which had their transected sciatic nerve repaired after delays of 1 day, 1 week, 1 month, and 2 months, respectively (called 1-day-delay, 1-week-delay, 1-month-delay, and 2-month-delay groups, respectively, in the following text). Half the rats in each group were electrically stimulated after the nerve repair (called ES rats in the following text), and the other half were sham-stimulated (called sham ES rats in the following text). After 5 days, 6 ES rats and 6 sham ES rats in each group were killed for Masson staining and quantitative PCR. The others were maintained for 6 weeks before the final functional tests and tissue observation.

### Surgical procedures

Surgical procedures were performed in a specific-pathogen-free animal laboratory using a microsurgical technique. All rats were anesthetized with sodium pentobarbital (30 mg/kg i.p.). After anesthesia, either side of the two limbs was treated in a sterile manner. The sciatic nerve trunk was cut sharply 1 cm proximal to the bifurcation to the common peroneal nerve and the tibial nerve. In order to prevent regeneration of the proximal nerve stump into the distal stump, it was turned around, embedded into the neighboring muscle, and secured by one 10-0 monofilament nylon stitch. The distal nerve stump was capped with a small polyethylene capsule. The four groups of animals were kept in their cages for specific delayed repair intervals before secondary repair surgery. The operated sciatic nerve stumps were re-exposed for secondary nerve repair surgery. The proximal end of each nerve was identified and freed from the neighboring muscle in which it had been embedded. The distal nerve stump was also identified and freed from the capsule. The tips of the proximal and distal nerve stumps were carefully trimmed which left a 5 mm nerve defect between proximal and distal nerve stumps. This nerve defect was bridged by a 1 cm chitin biologically absorbable conduit without tension [23]. Half the rats in each group were subjected to electrical stimulation. Two insulated copper wires were bared of insulation for 3–5 mm at their tips, which were to be used as electrodes. One tip of the two wires was twisted to form a loop, which was secured into the proximal end. The other tips of the wires were connected to an ES device. One hour of weak square 0.1 ms electrical pulses (3 V, 20 Hz) were then applied to the proximal nerve stumps. The other half was sham-stimulated. During ES and sham ES, the wound was covered by moistened paper to prevent the underlying tissues from drying out. After ES and sham ES, the wounds were closed again by layers. All the rats were retained to their cages. After 5 days, 6 ES rats and 6 sham ES rats in each group were killed for Masson staining and quantitative PCR. The other rats in each group were allowed to recover for 6 weeks before final functional tests and tissue processing.

### Masson staining

Five days after the secondary surgery, the nerves distal to the conduits were harvested for Masson staining. The longitudinal sections of the nerves were dewaxed and mordanted (10% potassium dichromate+10% trichloroacetic acid) for 30 min, nuclei were stained with hematoxylin for 20 min, differentiated with hydrochloric acid and ethanol for 15 s, returned to blue with weak ammonia for 15 s, stained with Masson solution (Cell Signaling Technology, Irvine, CA, U.S.) for 1 min, rinsed with 1% acetic acid, dehydrated with an increasing ethanol series, cleared with xylene I and II for 10 min to render sections transparent, and finally sealed in resin. Then collagen fiber proliferation was observed under a light microscope (Olympus, Tokyo, Japan) with the magnification of ×400. The images were analyzed with Image-pro Plus 5.0 software (Media Cybernetics, Shanghai, China).

### Quantitative PCR

Five days after the secondary surgery, L4–L6 spinal cords were harvested for 6 ES rats and 6 sham ES rats in each group. RNA was extracted using Trizol reagent (Invitrogen, Carlsbad, CA, U.S.). The purified RNA was treated with DNase using an RQ1 RNase-Free DNase kit (Promega, Madison, WI, U.S.). For the spinal cords, mRNA encoding BDNF and trkB receptor was reverse-transcribed to cDNA using a first-strand synthesis kit (Invitrogen). The amount of cDNA was quantified using real-time PCR. GAPDH quantification served as an internal control for normalization. The primers for these mRNAs are listed in Table 1. Relative differences in mRNA levels over control values were calculated using the ΔCt method according to the manufacturer's protocol (Applied Biosystems, U.S.). PCR reactions were independently repeated at least twice.

**Table 1:** Primer sequences for quantitative PCR.

Sample	Factor	Forward primer (5′→3′)	Reverse primer (5′→3′)
Spinal cord	BDNF	ACCATAAGGACGCGGACTTG	TGCCGCTGTGACCCACTC
Spinal cord	trkB receptor	GGCCAGATGCAGTGCTGAT	CATGCCTGCTGCGATTTG
Spinal cord	GAPDH	AGCAAGAGAGAGGCCCTCAGT	TTGTGAGGGAGATGCTCAGTCT

### Electrophysiological examination

Electrophysiological examination was conducted 6 weeks after the secondary surgery and before the animals were killed. After the rat was anesthetized with sodium pentobarbital (30 mg/kg body weight i.p.), the repaired sciatic nerve trunk was exposed, and stimulating bipolar electrodes were placed proximal and distal to the repair site in each group. The recording electrodes were placed in the gastrocnemius muscle, while the ground electrode was placed in subcutaneous tissue between the stimulating and recording electrodes. Rectangular pulses were used (duration 0.1 ms, 0.9 mA, 10 Hz, 6 continual stimuli). In order to determine the compound muscle action potential (CMAP) of each nerve, the intensity of the stimulation was gradually strengthened until the amplitude of the CMAP wave ceased to increase and a generally identical shape for the CMAP wave was formed from the stimulation at both the distal and proximal stumps. The peak amplitude and the latency of onset of CMAP were measured. The motor nerve conduction velocity (MNCV; m/s) was obtained semiautomatically by dividing the distance between the two stimulating sites by the difference in the conduction time.

### Osmium tetroxide staining

After electrophysiological examination, rats were deeply anesthetized with sodium pentobarbital (50 mg/kg body weight) and perfused through the left ventricle. A warm saline flush (250 ml) was followed by 250 ml of ice-cold 4% paraformaldehyde in 0.1 M pH 7.4 phosphate buffer. After perfusion, the entire nerve, including the repaired segment, was removed from each rat. Tissues were then harvested and fixed in 4% paraformaldehyde in 0.1 M phosphate buffer for 24 h at 4°C, stained in 1% osmium tetroxide for 12 h and then dehydrated through a graded series of ethanols, and the specimens were then immersed in xylene, embedded in paraffin, and sliced into 2 µm cross-sections. Images were acquired under light microscopy (Olympus, Tokyo, Japan), from which the total number of myelinated axons and myelin thicknesses and diameters were evaluated. Morphometric measurements were performed using Image-pro Plus 5.0 software (Media Cybernetics). The shortest lengths of the outer and inner margins of the myelin sheath were measured to determine the fiber diameter and axon diameter. Myelin thickness was calculated after the fiber and axon diameter were determined.

### Data analysis

All values are presented as the mean ± standard deviation (SD). Using SPSS 13.0 software, one-way analysis of variance (ANOVA) was used to compare the number of myelinated nerve fibers, the peak amplitude of CMAP, the latency of onset, the MNCV, and the morphometric measurements (including the fiber diameter, axon diameter, and myelin thickness) of the ES rats and sham ES rats in each group. *P*<0.05 was considered significant for all statistical comparisons.

## Results

### Axons counting

**Figure 1:**
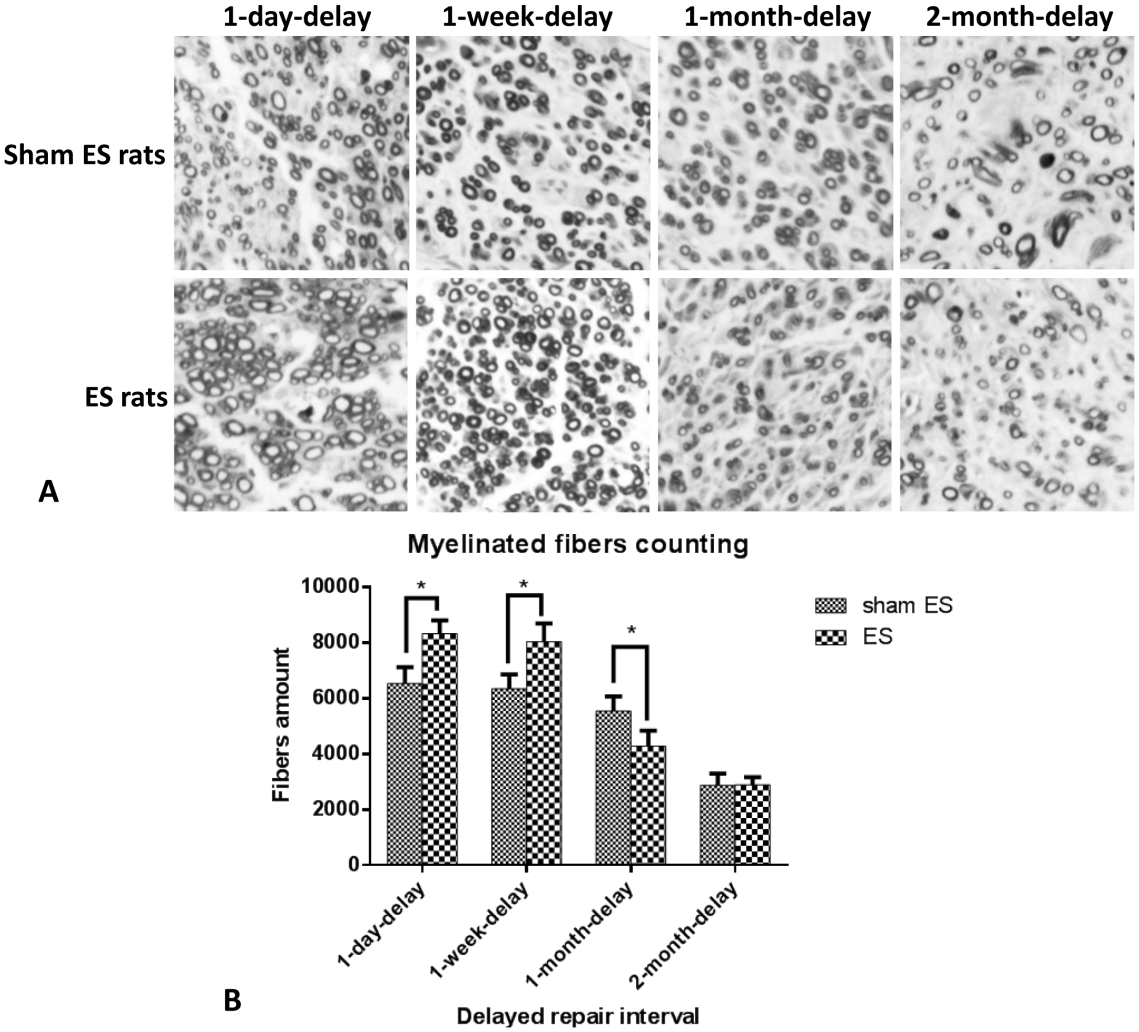
A. Osmium tetroxide staining of the myelinated fibers in distal nerve stumps. B) Count results of the myelinated fibers in the distal sciatic nerve stumps of the four groups of rats after 6 weeks of recovery. In each group, sham ES rats and ES rats were counted individually. There were more myelinated fibers in ES rats in both the 1-day-delay and 1-week-delay groups than among sham ES rats.

After 6 weeks of recovery, the regenerated axons in the distal nerve stumps were counted. The result was shown in Figure 1. The OsO_4_ stained fibers amounts in the distal nerve stumps of sham ES rats in 1-day-delay, 1-week-delay, 1-month-delay, and 2-month-delay groups were 6,525±593, 6,345±519, 5,549±524, and 2,873±422, respectively. For the ES rats, those values in corresponding groups were 8,324±472, 8,037±652, 4,276±559, and 2,895±269, respectively. In the 1-day-delay and 1-week-delay groups, there were more regenerated axons in ES rats than in sham ES rats (*P*<0.05). However, in the 1-month-delay groups, there were more axons in sham ES rats than in ES rats. In the 2-month-delay groups, there were no differences in the number of axons between the ES and sham ES rats. Higher axon counts indicated that electrical stimulation promoted collateral sprouting in the 1-day-delay group and 1-week-delay group. However, after 1 and 2 months of delay, electrical stimulation did not have any detectable effect on the axonal regeneration.

### Axons morphometric analysis

The fiber diameter and axon diameter were measured and the myelin thickness was calculated after 6 weeks of recovery. The results, shown in Table 2, indicate that these parameters differed between ES and sham ES rats in the 1-week-delay group. The fiber diameter, axon diameter, and myelin thickness of ES rats in 1-week-delay group were 1.93±0.09 µm, 0.96±0.14 µm, and 0.49±0.04 µm, respectively. In contrast, those parameters of sham ES rats in the same group were 1.62±0.06 µm, 0.76±0.08 µm, and 0.43±0.03 µm, respectively. The diameters of the fibers and axons and the myelin thickness of ES rats in the 1-week-delay group were visibly greater than those of sham ES rats in the same group. The fibers of ES rats in the 1-month-delay group also had greater diameters than those of the sham ES rats in the 1-month-delay group (1.76±0.15 µm vs. 1.63±0.10 µm).

**Table 2:** Fiber diameter, axon diameter, and myelin thickness of distal nerve stumps.

		Fiber diameter (µm)	Axon diameter (µm)	Myelin thickness (µm)
1-day-delay	Sham ES	1.76±0.17	0.81±0.14	0.48±0.05
	ES	1.71±0.12	0.80±0.16	0.46±0.06
1-week-delay	Sham ES	1.62±0.06	0.76±0.08	0.43±0.03
	ES	1.93±0.09*	0.96±0.14*	0.49±0.04*
1-month-delay	Sham ES	1.63±0.10	0.76±0.14	0.43±0.05
	ES	1.76±0.15*	0.82±0.07	0.47±0.08
2-month-delay	Sham ES	1.97±0.13	1.09±0.08	0.44±0.05
	ES	2.00±0.14	1.06±0.11	0.47±0.07

*P<0.05 versus sham ES rats in the same group.

### Measurement of MNCVs and CMAPs

**Figure 2:**
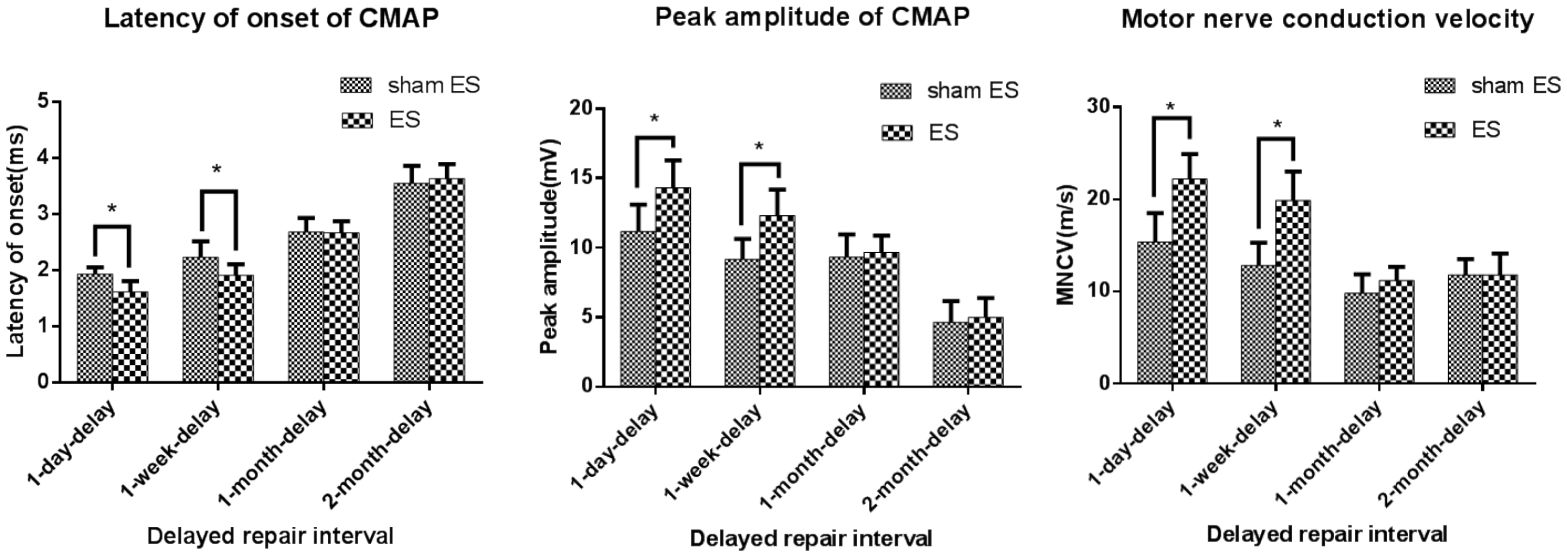
Measurement of peak amplitude, onset of latency of CMAP and MNCV of sham ES rats and ES rats in the four groups. Differences were found between sham ES rats and ES rats in the 1-day-delay and 1-week-delay groups with these respects.

In order to evaluate the conduction properties of the operated sciatic nerves, electrophysiological examination was performed after 6 weeks of recovery. The results are shown in Table 3 and Figure 2. The peak amplitudes of ES rats in 1-day-delay and 1-week-delay groups were higher than those of sham ES rats in corresponding groups (*P*<0.05). For the latency of CMAP onset, there were differences between the sham ES rats and ES rats in 1-day-delay group and 1-week-delay groups. The latencies of onset in ES rats of these two groups were shorter than those of sham ES rats (*P*<0.05). The motor nerve conduction velocities (MNCVs) were calculated using CMAP properties. After 6 weeks of recovery, the MNCVs of sham ES rats in 1-day-delay, 1-week-delay, 1-month-delay, and 2-month-delay groups were 15.4±3.1 m/s, 12.8±2.5 m/s, 9.8±2.1 m/s, and 11.8±1.7 m/s, and those of ES rats in corresponding groups were 22.2±2.7 m/s, 19.9±3.1 m/s, 11.2±1.5 m/s, and 11.8±2.3 m/s. In 1-day-delay and 1-week-delay groups, the MNCVs of ES rats were greater than those of sham ES rats. However, in 1-month-delay and 2-month-delay groups, there were no differences between the MNCVs of ES rats and sham ES rats.

**Table 3:** Peak amplitude, latency of onset of compound muscle action potential, and motor nerve conduction velocity in each group.

		Peak amplitude (mV)	Latency of onset (ms)	MNCV (m/s)
1-day-delay	Sham ES	11.17±1.94	1.93±0.12	15.4±3.1
	ES	14.33±1.97*	1.62±0.19*	22.2±2.7*
1-week-delay	Sham ES	10.00±0.89	2.23±0.28	12.8±2.5
	ES	12.33±1.86*	1.92±0.19*	19.9±3.1*
1-month-delay	Sham ES	9.33±1.63	2.68±0.25	9.8±2.1
	ES	9.67±1.21	2.67±0.21	11.2±1.5
2-month-delay	Sham ES	4.67±1.51	3.55±0.31	11.8±1.7
	ES	5.00±1.41	3.63±0.25	11.8±2.3

*P<0.05 versus sham ES rats in the same group.

### Masson staining

**Figure 3:**
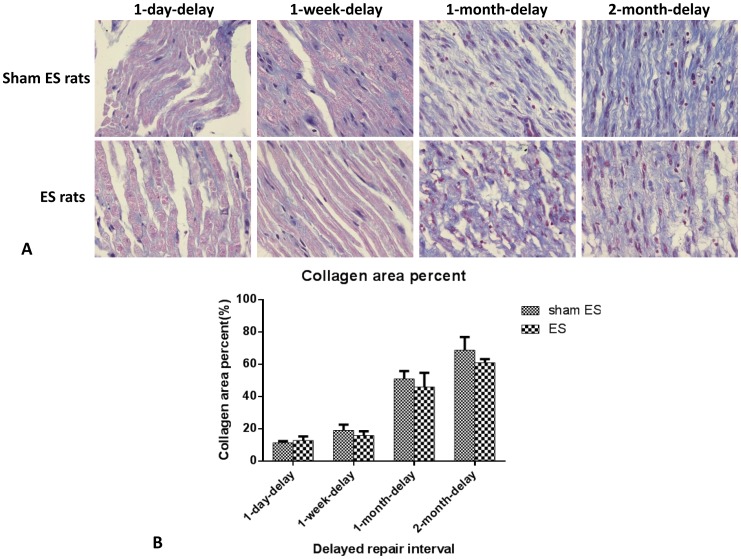
A. Masson staining of the distal nerve stumps of the four groups of rats five days after the secondary surgery. One month after the secondary surgery, the myelin structure was completely gone, and there was much more collagen tissue hyperplasia. B. Areas of scar tissue for sham ES and ES rats in the four groups. Areas were automatically calculated using Image-pro Plus 5.0 image analysis software.

Five days after the secondary surgery, the nerves distal to the conduits were harvested for Masson staining. Results are shown in Figure 3. In the 1-day-delay and 1-week-delay groups, the collagen tissue hyperplasia was not great for either ES rats or sham ES rats. The nerve fibers were lined in order, with few collagen fibers and orderly arrangement. However, in the 1-month-delay and 2-month-delay groups, both ES rats and sham ES rats completely lacked myelinated nerve fibers. There were more collagen fibers and they were disordered. Using the Image Pro software, the scarred area was measured, as shown in Figure 3B. In the 1-day-delay and 1-week-delay groups, distal nerves made up no more than 20% of the total area. However, in the 1-month-delay and 2-month-delay groups, they made up approximately 50%, which indicated that collagen tissue hyperplasia was still significant after chronic denervation of distal nerves after 1 month or more.

### Quantitative PCR

**Figure 4:**
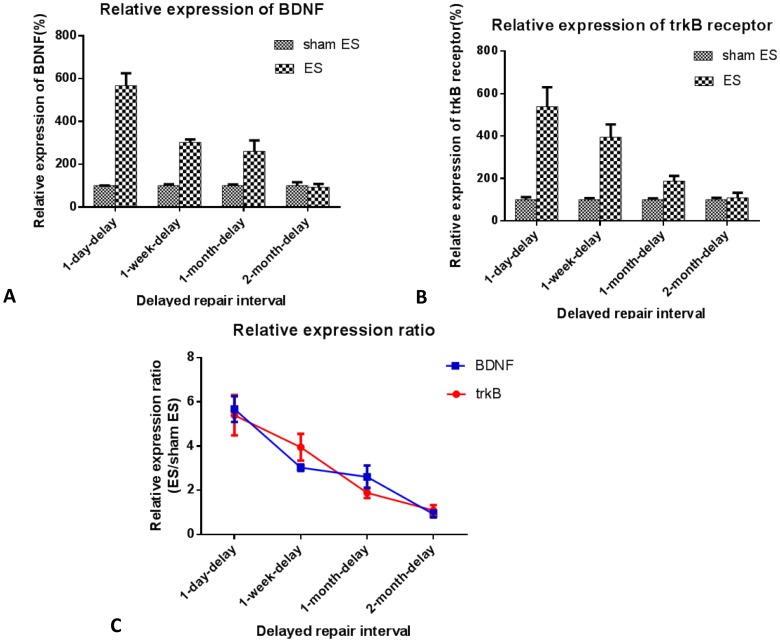
Effect of electrical stimulation on BDNF and trkB receptor expression in L4–6 spinal cord neurons. A. BDNF and B. trkB receptor mRNA expression was quantified using RT-PCR. Values are shown as means±SEM normalized to GAPDH from at least three independent trials. The ratios of relative expressions of BDNF and trkB receptor of ES rats to those of sham ES rats were plotted in trend curves (C).

Five days after the secondary surgery, the L4–6 spinal cords were harvested for evaluation of the expression levels of BDNF and trkB receptor in the neurons. Results are shown in Figure 4. Quantitative PCR showed that the relative transcription levels of BDNF and trkB mRNA of ES rats in the 1-day-delay group were 5.7 and 5.4 times of those of sham ES rats, respectively. The ratio of relative transcription levels of these two factors of ES rats to those of sham ES rats decreased as the delay interval increased. In the 2-month-delay group, the transcription levels of ES rats were nearly the same as those in sham ES rats (ES rats/sham ES rats: 0.9 for BDNF, 1.1 for trkB receptor). Results indicated that, after chronic axotomy of proximal nerves, the effects of electrical stimulation on the expression promotion of nerve growth factors in the neurons decreased.

## Discussion

During peripheral nerve injury, complex pathophysiologic changes, including morphologic and metabolic changes happens at the site of injury and the cell bodies in as little as a few hours. The proximal nerve stump degenerates up to the first node of Ranvier and the growth cones sprout many axons, which grow toward the distal nerve stump. It takes the axons a long time to cross the gap between the stumps, after which the regenerated axons proceed at a rate of 1–3 mm/d in the distal nerve stump. In rats, a period of about 1 month transpires before all motoneurons regenerate their axons across a lesion site. The long delay caused by the axonal outgrowth across the lesion site is one major cause of poor functional recovery. This is because the Schwann cell environment in the distal nerve stump deteriorates over time. In previous studies, 1 h of weak electrical stimulation was found to promote nerve regeneration across the gap, which shortened the time of axonal regrowth across the lesion site and into the target muscles [14]. However, such experiments were mostly performed right after nerve transection. In clinical cases, most patients with total or partial nerve axotomy were treated between 72 h and 7 d after injury [24]. In some special cases, such as those in which the wound is contaminated or the patients had some underlying disease, nerve repair surgery must be delayed until the other problems are controlled. Whether electrical stimulation remains effective as a means of fostering peripheral nerve regeneration after delayed repair, and after how long of a delay, remains to be determined.

In this study, a delayed nerve repair model was used to confirm whether electrical stimulation had any effect on nerve regeneration after delayed repair. The myelinated fibers were counted after 6 weeks of recovery. In general, with longer delays in repair, there were fewer myelinated fibers in both the ES and sham ES rats. The reduction in the number of myelinated fibers in the distal nerve stump was readily visible in ES rats after a delay of 1 month and in sham ES rats after a delay of 2 months. The last result has been confirmed in many studies. One previous study showed that short-term denervation lasting ≤4 weeks did not affect axonal regeneration but more prolonged denervation profoundly reduced the numbers of backlabeled motor neurons and axons in the distal nerve stump [13]. In 3- and 6-month-delay repair samples, the number of Schwann cells declined in the distal nerve segments but fibrosis and proteoglycan scarring increased, all of which interferes with axonal regeneration [21]. There were more myelinated fibers in ES rats than in sham ES rats in the 1-day-delay and 1-week-delay groups. However, in the 1-month-delay group, the ES rats had fewer regenerated axons growing into the distal stump than the sham ES rats. The motor nerve conduction properties of sham ES rats and ES rats in the four groups were consistent with fiber counts. The results of peak amplitude and latency of onset of CMAP and MNCVs of ES rats in the 1-day-delay group and 1-week-delay group were better than those of sham ES rats in corresponding groups. However, no differences were observed between ES and sham ES rats in the 1-month-delay or 2-month-delay groups. So it could be concluded that the effective period for electrical stimulation's promotion of nerve regeneration after delayed repair was no longer than 1 month.

The poor effect of electrical stimulation on nerve regeneration after delays of 1 month or more may be correlated with two factors. First, the neurons of peripheral nerves in the spinal cord and dorsal root ganglion may die during the delay because of the loss of nutrition from the distal target. A number of studies have demonstrated that different neurotrophic factors, such as BDNF [25]–[27], neurotrophin-3 [28], and neurotrophin-4/5 [29] can rescue adult spinal motoneurons from retrograde degeneration after ventral root avulsion, restore their cholinergic phenotype after peripheral nerve transection, and stimulate motor axonal regeneration. The motoneurons were found to survive and express a series of growth-associated proteins. This was found to be the mechanism by which electrical stimulation promoted axonal regeneration [17], [18]. However, the number of fast-blue stained neurons decreased 6–8 weeks after the delayed nerve repair took place [30], [31]. Activation of transcription factor 3′ expression, a marker of nerve injury, was impaired after a 1-month delay in motoneuron repair and a 2-month delay in sensory neuron repair [32]. In the current experiment, the transcription levels of BDNF and trkB receptor in the neurons of the spinal cord were measured shortly after nerve repair with or without electrical stimulation. Results showed that the relative expression levels of BDNF and trkB of ES rats were different from those of sham ES rats after delayed repair. In 1-day-delay, 1-week-delay, 1-month-delay, and 2-month-delay groups, the relative expression levels of BDNF of ES rats were 5.7 times, 3 times, 2.6 times, and 0.9 times those of sham ES rats in corresponding group, and the relative expression levels of trkB receptor of ES rats were 5.4 times, 3.9 times, 1.9 times, and 1.1 times those of sham ES rats in corresponding groups. With longer delays, the effects of electrical stimulation on the promotion of the expression of BDNF and trkB in the neurons decreased. Second, Schwann cells in the distal nerve stump underwent apoptosis after long-term denervation [33], and chronically denervated Schwann cells may be less able to respond to axonal signals than their acutely denervated counterparts [34]. Masson staining results in this experiment showed that in 1-day-delay and 1-week-delay groups, nerve fibers were nearly intact morphologically. The pink-stained myelin was uninterrupted and plump. However, in the 1-month-delay and 2-month-delay groups, the lined structures of the myelinated fibers disappeared. Collagen tissue hyperplasia was apparent. The Schwann cells were scarcely distributed among the collagen tissue. Associated with impaired regeneration after long-term Schwann cell denervation, growth factors such as glial cell-line-derived neurotrophic factor (GDNF) showed less expression [35]. Deterioration of the Schwann cell environment after long delays in repair was found to be disadvantageous to nerve regeneration, which showed no differences between the ES rats and sham ES rats in 1-month-delay and 2-month-delay groups with respect to axon count and motor nerve conduction velocity.

Previous studies have addressed the effects of electrical stimulation on nerve regeneration after delayed repair. In a study by Jinghui Huang et al., the sciatic nerve of SD rats was transected, and the repair of nerve injury was delayed for different periods [36]. Brief ES was applied to the proximal nerve stumps when the transected nerve stumps were bridged with hollow nerve conduits after delays. Electrical stimulation was found to promote nerve regeneration after delays in repair, with the longest lasting 24 weeks. However, current results showed that the effective period for electrical stimulation after delayed nerve repair was less than one month. The differences between Huang's results and the current report may be related to the following factors. First, different animal models were used. In the Huang experiment, the sciatic nerve was transected, and the two stumps were deflected and sutured to nearby muscles. In the current experiment, the distal nerve stump was capped in a capsule. It is here hypothesized that the embedment of nerve stump into muscle may have some negative effect on the degeneration progress of distal nerve. However, no proof of this hypothesis was found in the current work. Second, the conduits used in two experiments were different, which may be one cause of the differences in the experimental results. The third reason is that the means and time of electrical stimulation were different. In the Huang experiment, one electrode was placed around the proximal nerve stump, and another electrode was placed on a muscle close to the nerve. Then 20 min of electrical stimulation was used on each rat. In the current experiment, the two electrodes were placed around the proximal nerve stump. Electrical stimulation in our experiment lasted 1 h. In this way, the transmission of electric fields differed between the two experiments. The effects of electrical stimulation on nerve regeneration may differ. Another paper discusses the effects of electrical stimulation on nerve regeneration after carpel tunnel syndrome [37]. Participants were found to suffer from carpel tunnel syndrome for different periods of time. The results of this clinical trial showed that the stimulation group had significant axonal regeneration 6–8 months after the carpel tunnel release surgery. However, the focus of this clinical trial was carpel tunnel patients. The median nerves were compressed but not transected. There were no neural defects in these patients. Outgrowth of axonal sprouts is more rapid after crushing injuries in which the continuity of the nerve sheaths and basement membrane is preserved than after nerve section [38], [39].

## Conclusion

Electrical stimulation has been shown to be an effective means of promoting nerve regeneration after nerve injury. In this study, a delayed nerve repair model was designed and electrical stimulation was applied after delays in repair. Results showed that electrical stimulation had an effect on nerve regeneration in delays in repair lasting less than 1 month. This effect was proven to be related to two factors. First is the expression regulation of neurotrophic factors by electrical stimulation. After chronic axotomy of proximal nerves, the effects of electrical stimulation on the expression of nerve growth factors in the neurons decreased. Second is the regeneration environment in the distal nerve. Collagen tissue hyperplasia was found to be significant after chronic denervation of distal nerves after 1 month or more. This may contribute negatively to the axonal regeneration in the distal nerve.
